# Preparation of Dissolving Pulp by Combined Mechanical and Deep Eutectic Solvent Treatment

**DOI:** 10.3390/polym15163476

**Published:** 2023-08-19

**Authors:** Xincai Li, Jiachuan Chen, Baobin Wang, Lei Zhang, Kai Zhang, Guihua Yang

**Affiliations:** 1 State Key Laboratory of Biobased Material and Green Papermaking, Qilu University of Technology, Shandong Academy of Sciences, Jinan 250353, China; 2 Guangxi Key Laboratory of Clean Pulp & Papermaking and Pollution Control, College of Light Industry and Food Engineering, Guangxi University, Nanning 530004, China

**Keywords:** deep eutectic solvent, cellulose pulp, mechanical treatment, acid DES pulping

## Abstract

Grasses are potential candidate to replace wood as a raw material for pulping and paper making, and several processes have been developed to produce grass pulp. In this study, wheat straw was used as raw material, and the possibility of sequential treatment with a mechanical method and deep eutectic solvent (DES) to prepare high-quality dissolving pulp was explored. Firstly, the wheat straw was mechanically treated, and then the wheat straw was delignified using a choline chloride–lactic acid deep eutectic solvent. The results showed that the optimal treatment conditions of deep eutectic solvent were 110 °C, 6 h, and a solid–liquid ratio (ratio of pulp to DES) of 1:40. The removal rate of lignin was 82.92%, the glucose content of pulp was increased by 11.42%. The DES recovery rate was further calculated, and the results showed that the DES recovery rate was more than 50% with rotary evaporation. The pulp viscosity after bleaching was 472 mL/g, and the α-cellulose accounted for 81.79%. This treatment has advantages in biomass refining, and the total utilization rate of wheat straw reaches 72%. This study confirmed that combined mechanical and deep eutectic solvent treatment can effectively remove lignin from wheat straw to produce high-quality wheat straw dissolving pulp.

## 1. Introduction

The rapid development of the economy has resulted in an increasing demand for non-renewable energy sources such as coal, oil, and natural gas. However, this trend has led to issues such as energy resource scarcity and environmental pollution [1,2]. Therefore, the development of renewable energy and the promotion of green reagents have garnered significant attention [3]. Biomass resources are attracting increasing attention due to their renewable nature and extensive source [4]. Nevertheless, the reutilization rate of agricultural and forestry residues remains significantly low [5,6]. A significant amount of straw undergoes natural degradation or is inadequately utilized, with some being burned in the open air, resulting in air pollution [7]. The current relatively mature pulping process includes mechanical pulping and forms of chemical pulping such as alkali pulping or sulfite pulping, but both of them have certain defects, such as the low lignin-removal rate in mechanical pulping and the production of a large amount of waste liquid and the release of harmful gases such as sulfur dioxide (H_2_S), methyl mercaptan (CH_4_S), and dimethyl sulfide (C_2_H_6_S) in chemical pulping [8,9].

A deep eutectic solvent (DES) is a new type of ionic solvent system first discovered by Abbott et al. [10]. It is mainly synthesized using a hydrogen bond donor (HBD) and hydrogen bond acceptor (HBA), and it has a lower melting point than a single component. It also has the characteristics of low cost, easy preparation, and high recyclability [11]. In recent years, DESs have shown potential application value in bio-catalysis [12], natural product extraction [13], electrodeposition [14], material synthesis [15], and extraction of bioactive components [16]. Like ionic liquids, DESs also have a good delignification effect. Francisco et al. demonstrated that lactic acid/choline chloride (10:1) exhibits favorable delignification selectivity, with high solubility of lignin and low solubility of cellulose in the solvent [17]. In addition, DESs also play a role in the degradation of hemicellulose [18]. Dugoni et al. [19] used choline acetate (ChOAc) as a hydrogen bond acceptor (HBA) and glycolic acid (GlyA), levulinic acid (LevA), and imidazole (Im) as hydrogen bond donors (HBDs) to prepare three novel deep eutectic solvents (DESs); it was found that after ChOAc:LevA treatment, lignin was completely removed, and the hemicellulose decreased by 40–50%. Duan et al. [20] investigated the effects of acidic, alkaline, and neutral DESs on the lignin-removal performance of bamboo dissolving pulp. The choline chloride/oxalic acid acidic DES system has the best delignification effect because many activated protons (H^+^) in the acidic DES can destroy the fiber structure effectively and the addition of the acidic DES promotes the swelling of the fiber, which in turn improves accessibility. Under the DES system, the optimal reaction conditions for delignification are 60 °C and 30 min, and the solid–liquid ratio is 1:10. The treatment temperature and treatment time have a great influence on the removal rate of lignin. Guo et al. [21] found that the removal rate of lignin increased with the increase in temperature. In addition, when a certain temperature is reached, the DES treatment time has little effect on the removal effect of lignin. Compared with ionic liquids, the recovery of a DES is easier, which can effectively reduce costs. Duan et al. [22] used microwave-assisted deep eutectic solvents to enhance the performance of bamboo dissolving pulp. The polarization, rotation and vibration of DES ions/molecules and temperature increase caused by microwaves can further promote fiber swelling and cellulose degradation and successfully reduce pulp viscosity and increase Fock reactivity. At present, a variety of DESs have been successfully used to treat wheat straw. Guo et al. [23] used a choline chlorine–oxalic acid system to treat wheat straw, proving that DES treatment can significantly improve the durability and calorific value of wheat straw residue particles. Yue et al. [24] successfully isolated lignin from wheat stalks using a K_2_CO_3_-glycerol system. Lou et al. [25] treated wheat straw with a choline chlorine–lactic acid system to produce lignin nanoparticles, and the study proved that the system could extract high-purity lignin (94.8%) from wheat straw. Then, Suopajarvi et al. [26] treated wheat straw raw materials with five acidic DESs and one basic DES. It was found that the choline chloride–lactic acid system had a good effect on lignin removal.

In this paper, a combination of mechanical treatment and the choline chloride/lactic acid deep eutectic solvent system was employed for co-processing. Firstly, the optimal DES treatment conditions for wheat straw pulp, including reaction temperature, reaction time, and solid–liquid ratio, were explored. Subsequently, the physical and chemical properties of the treated dissolving pulp and its components were analyzed, and the DES was successfully recovered. This work demonstrates the possibility of mechanical and DES sequential treatment to produce dissolved pulp of wheat straw. The first stage of mechanical pretreatment facilitates the accessibility of solvent to wheat straw and provides the possibility for the preparation of higher-quality dissolved pulp.

## 2. Materials and Methods

### 2.1. Materials

Wheat straw was obtained from Jinan, Shandong. Choline chloride (98%), lactic acid (AR), and all chemicals were used as received without further purification. All the water used in the experiment was deionized water made by the laboratory.

### 2.2. Wheat Straw Raw Material and Preparation of DES

First, mechanical treatment of wheat straw was carried out; the specific steps were as follows: The wheat straw was washed with clean water, the impurities such as wheat residue were removed, and the air-dried wheat straw was dried at 40 °C for 48 h. The dried wheat straw was treated three times using a single-screw extruding and dredging machine, followed by a disk mill for three stages of grinding, and the grinding tooth spacing was 0.5 mm, 0.3 mm, and 0.15 mm, respectively. After grinding, the slurry was submerged in warm water at 60 °C for 10 min. Finally, air-dried mechanical pulp (DMP) was obtained by air-drying at room temperature. The DES was obtained by mixing choline chloride and lactic acid in a glass vial with a molar ratio of 1:10 and stirring magnetically at 60 °C and 200 r/min for 2 h. Choline chloride is a hydrogen bond acceptor (HBA), and lactic acid is a hydrogen bond donor (HBD) [27].

### 2.3. Optimization of DES Processing Conditions

#### 2.3.1. Different Temperatures

The wheat straw pulp was subjected to treatment at different temperatures: 80 °C, 110 °C, 140 °C, and 160 °C. The specific operational procedure was as follows: One g of absolute-dried pulp and 40 g of deep eutectic solvent (DES) were placed into a 100 mL thick-walled pressure-resistant bottle and heated at the respective temperature (80 °C, 110 °C, 140 °C, or 160 °C) for 6 h. After the reaction, the mixture was centrifuged at 10,000 rpm for 10 min, and the resulting solution was filtered through a G2 glass filter. The solid residue remaining on the filter was washed with 50 mL of ethanol, followed by hot water until the filtrate became colorless. The solid residue was then air-dried in an oven at 45 °C. Meanwhile, the filtrate was transferred to a 2000 mL beaker, and 10 times the volume of water was added. The mixture was refrigerated overnight [28]. 

#### 2.3.2. Different Solid–Liquid Ratios

Wheat straw pulp was treated under the conditions of DMP–DES mass ratio of 1:20, 1:40, 1:60, and 1:80 and reacted at 110 °C for 6 h. After the reaction, the operation was the same as above.

#### 2.3.3. Different Times

The wheat straw pulp was treated at different reaction times of 0.5 h, 1 h, 2 h, and 4 h, under the condition of 110 °C. For each reaction, 1 g of absolute dry DMP and 40 g of DES were placed into the reactor. After the reaction, the subsequent operations were carried out as described earlier.

### 2.4. Lignin Recovery and DES Regeneration

Ten times the volume of water was mixed with the resulting filtrate, refrigerated overnight, filtered out by a G3 glass filter, dried in a vacuum environment of 45 °C for 6 h, and weighed [29]. The filtrate was treated in a rotary evaporator to remove water and ethanol from the filtrate, and the obtained liquid was the regenerated DES (R-DES). 

The content of lactic acid, glucose, and xylose in DES recovered by rotary evaporation was detected by high-performance liquid chromatography; the mobile phase was 250 mmol/L H_2_SO_4_ solution, the flow rate was 0.6 mL/min, and the detection time was 20 min. When calculating the recovery of DES, 1 g of lactic acid is equivalent to 1.155 g of DES. The formula for the calculation of the DES recovery rate is as follows [28]:

(1) $$DES\;recovery\;rate(\%)=1.155*\frac{c*V}{m}*100\%$$

In the formula, *c* is the content of lactic acid, g/L; *V* is the volume of DES obtained after rotary evaporation, L; and *m* is the initial weight of DES, g.

The lignin content in the wheat straw fiber was determined using the NREL method in the aforementioned G2 glass dryer [30]. First, 0.30 g (±0.01 g) (*m*_0_) of wheat straw fiber was weighed and placed into a 100 mL digestion tube. Then, 3.0 mL of 72% H_2_SO_4_ solution was added, shaken well, and incubated in a constant temperature water bath at 30 °C for 1 h, with shaking every 10 min during this period. Once the heat preservation was completed, it was transferred to a pressure bottle. Then, 84 mL of distilled water (diluted to 4%) was added, and it was incubated in an autoclave at 121 °C for 1 h. The dry G2 glass sand core filter was taken and weighed as *m*_1_, the above-mentioned acid-dissolved lignin was filtered, the filter residue was put in an oven at 105 °C and dried to constant weight (*m*_2_), and the content of acid-insoluble lignin (AIL) was calculated. The filtrate was retained for analysis and detection of cellulose components.

The formula for calculating the acid-insoluble lignin content X in wheat straw fiber is as follows:

(2) $$X=\frac{m_{1}-m_{2}}{m_{0}}\times 100\%$$

### 2.5. Pulp Bleaching

The pulp after DES treatment was bleached using a hypochlorite and hydrogen peroxide (H&P) two-stage bleaching method. The detailed conditions were as follows:

H: pulp concentration 6%, effective chlorine dosage 7.0%, NaOH dosage 0.5%, treatment temperature 40 °C, treatment time 90 min.

P: slurry concentration 8%, H_2_O_2_ dosage 7%, NaOH dosage 0.5%, processing temperature 80 °C, processing time 90 min.

### 2.6. Characterizations 

#### 2.6.1. SEM

The freeze-dried sample was fixed on a metal sample table with conductive adhesive. Before observation, the freeze-dried samples were coated with gold using a vacuum sputter coater. The surface morphology of the sample was observed using a benchtop scanning electron microscope (EM-30 Plus+). The acceleration voltage was 5 kV. All the photos were enlarged by a factor of 200.

#### 2.6.2. FT-IR

The functional groups of raw materials and DES-treated slurry were analyzed using a Fourier transform infrared spectrometer (ATR, Bruker ALPHA, Germany). The scanning range was from 700 cm^−1^ to 4000 cm^−1^, and the resolution was 4 cm^−1^. The scanning time was 32 s.

#### 2.6.3. XRD

The crystallinity of the sample was determined using an X-ray diffractometer (XRD, D8-ADVANCE, Bruker, Germany). The scanning angle was 10° to 40°; the scanning step was 20.0°/min. The working voltage and current were 40 kV and 40 mA, respectively. Crystallinity (CrI) was calculated according to Formula (3) [31]:

(3) $$CrI=\frac{I_{002}-I_{am}}{I_{002}}\times 100\%$$

In the formula, *I*_002_, representing the peak intensity of the crystalline region, is the maximum peak intensity at a 2θ angle close to 22.6° and *I_am_*, representing the peak intensity of the amorphous region, is the minimum diffraction intensity at a 2θ angle close to 15.8°.

#### 2.6.4. UV-Vis

A certain amount of acid hydrolysis solution was taken out and placed in a 50 mL centrifuge tube. Then, it was centrifuged and filtered. The filter residue was dried to constant weight. The filtrate was tested according to GB/T 2677.8-1994 [32]. The filtrate was diluted to an appropriate multiple, a UV spectrophotometer was used to detect the absorbance value of the sample at 205 nm, and the acid-soluble lignin (ASL) content was calculated as shown in the following formula:

(4) $$B=\frac{A*D}{110}$$

In the formula, *B* is the lignin content in the filtrate, g/L; *A* is the absorbance value of the diluted test solution sample at 205 nm; *D* is the dilution factor of the sample; and the number 110 is the absorbance coefficient, L/(g·cm^−1^).

The total lignin mass in the acid hydrolysis liquid includes dissolved lignin and lignin precipitates in the liquid to be tested. Therefore, the formula for calculating the total lignin content in the acid hydrolysis solution is as follows:

(5) $$M=M_{1}+B*V$$

In the formula, *M* is the quality of the total lignin in the acidolysis solution, g; *M*_1_ is the quality of the filter residue, that is, the lignin precipitate in the acidolysis solution, g; *B* is the content of the filtrate, that is, the lignin dissolved in the acidolysis solution, g/L; and *V* is the volume of the acid solution, L.

#### 2.6.5. Cellulose Component Analysis

The polysaccharides in the samples were hydrolyzed into monosaccharides using the NERL method, and then the content of monosaccharides was determined. Sugar components in the hydrolyzed solution were quantitatively determined by ion chromatography (ICS 5000+). A CarboPac PA 20 analytical column was used to separate the sugar in the sample in the form of monomers. The mobile phase was 50 mmol·L^−1^ sodium hydroxide solution and ultrapure water, the flow rate was 0.4 mL·min^−1^, and the elution solid and liquid proportions were 4% and 96%, respectively.

#### 2.6.6. Pulp Property Analysis

A Kaiser fast sheet former was used to make paper from the unbleached and bleached pulp. The weight of the paper was 60 g/m^2^. Each sample was measured three times in parallel, and the average value was recorded as the final whiteness value. Bleached pulp viscosity was measured using an automatic pulp viscometer (Viscomat, Lagge Control AB, Sundavall, Sweden) according to Tappi T230 om-99 using the copper ethylenediamine (CED) method. The bleached paper sheet was torn into pieces of about 1 × 1 cm, and the weight of α-cellulose in the pulp was measured according to TAPPI203 [33]. The Fock reactivity test was conducted according to the method reported by Tian et al. [34]. The fiber morphology of the pulp was analyzed using a fiber quality analyzer (Valmet FS5, Valmet, Espoo, Finland).

## 3. Results and Discussion

### 3.1. Effect of Mechanical Treatment on Pulp Components

**Figure 1:**
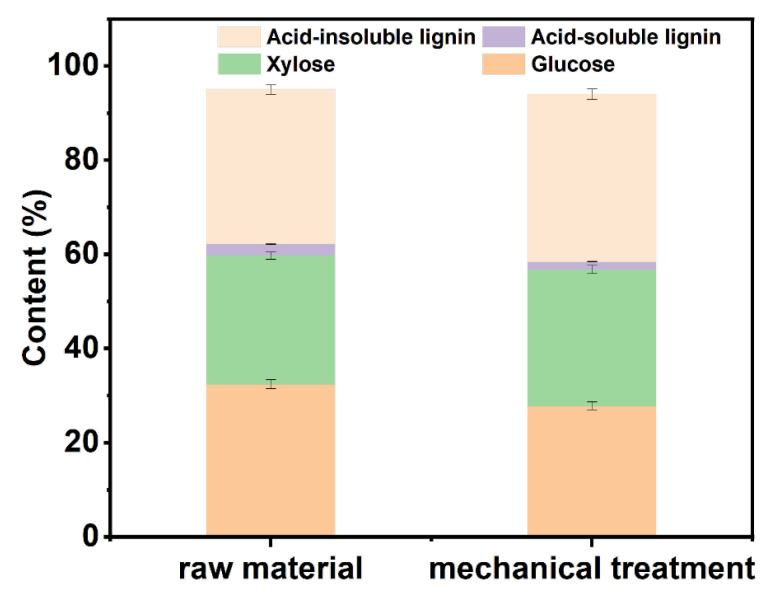
Component analysis chart of pulp after raw material and mechanical treatment.

As depicted in Figure 1, mechanical treatment is accompanied by thermal elevation which would lead to the degradation of carbohydrates in the raw material. Meanwhile, the lignin removal during this stage is minimal. Thus, the ratio of glucose content decreased, and the ratio of lignin in the mechanically treated material increased. The yield of mechanical treatment of wheat straw is 93.97%. Mechanical treatment can expose more fibers, thereby increasing the contact area between fibers and chemical solution and enhancing the delignification effect of the DES in the subsequent treatment process [35].

### 3.2. Optimization of Processing Conditions Using the OFAT Method

#### 3.2.1. Effect of Processing Temperature

**Figure 2:**
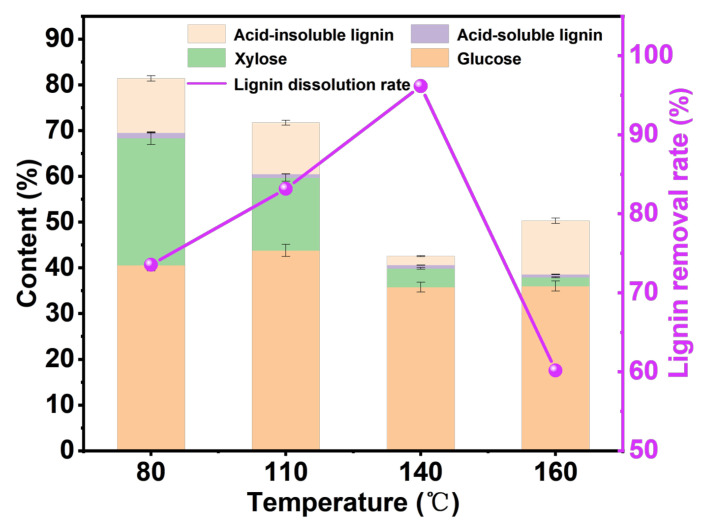
The analysis chart of pulp components at different reaction temperatures under the reaction time of 6 h and the mass ratio of wheat straw to DES of 1:40.

As shown in Figure 2, under the reaction time of 6 h and the mass ratio of wheat straw to DES of 1:40, the effect of treatment temperature on the content of each component was analyzed. The glucose content reaches the highest level (43.80%) at 110 °C, while at 140 °C and 160 °C, the cellulose content is low, which is due to the severe degradation of cellulose at temperatures above 140 °C [36]. It should be noted that when the temperature reaches 160 °C, the lignin-removal rate decreases, which may be caused by the dehydration and condensation of polysaccharides at high temperatures to form lignin-like substances [37]. From 80 °C to 140 °C, the removal rate of lignin increases with the increase in temperature, reaching 96.45% at 140 °C. The pulp yields at 140 °C and 160 °C were lower than those at 80 °C and 110 °C, and the results were similar to those of previous studies [38]. Due to the higher pulp yield and higher glucose content at 110 °C, we next explored the effect of treatment time on the content of each component of pulp at 110 °C. 

#### 3.2.2. Effect of Processing Time

**Figure 3:**
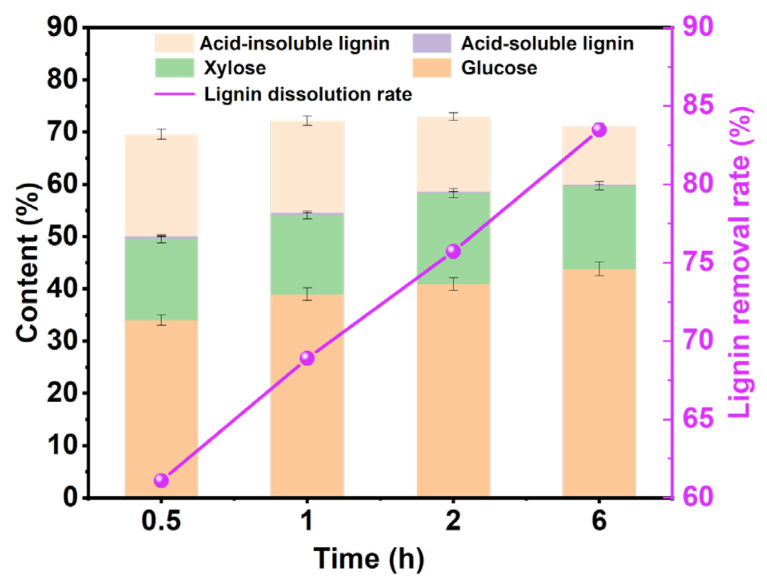
The analysis chart of pulp components under different reaction times under the reaction time of 6 h and the solid-to-liquid ratio of 1:40.

According to the foregoing determination, the optimal reaction temperature is 110 °C. As shown in Figure 3, under the conditions of a reaction temperature of 110 °C and a reaction solid-to-liquid ratio of 1:40, the influence of different reaction times on the content of pulp components was explored. As the reaction time increased, the proportion of glucose showed an upward trend, which indicated that with the increase in reaction time, the proportion of cellulose in pulp gradually increased. At the same time, with the increase in reaction time, the lignin-removal rate gradually increased, indicating that increasing the reaction time can improve the lignin-removal effect of DES, which is consistent with previous research [39]. Therefore, we will explore the influence of different solid–liquid ratios on the content of pulp components at a reaction time of 6 h and a reaction temperature of 110 °C. 

#### 3.2.3. Effect of Solid–Liquid Ratio

**Figure 4:**
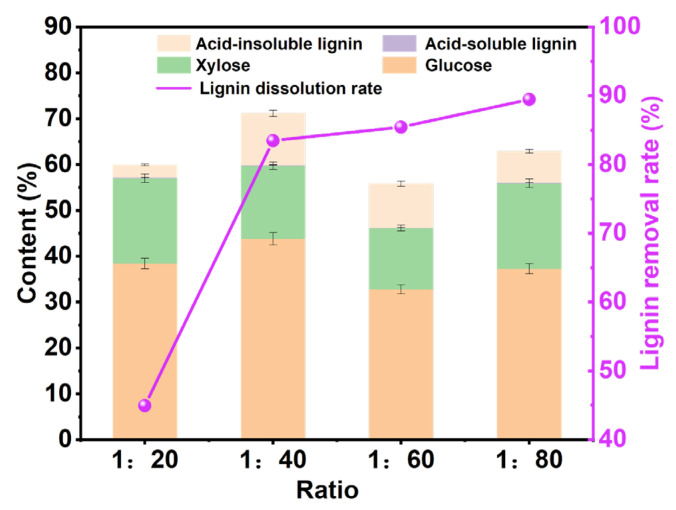
The analysis chart of pulp components processed at different solid–liquid ratios under the reaction time of 6 h and the reaction temperature of 110 °C.

According to the above research, we determined the reaction temperature and reaction time as 110 °C and 6 h. Moreover, a suitable solid–liquid ratio is a necessary condition to ensure that the pulp is treated completely, and a lower solid–liquid ratio will make the pulp be treated inadequately, thus affecting the performance of the pulp. As shown in Figure 4, the influence of different solid–liquid ratios on the content of different components of pulp was explored. With the increase in the solid–liquid ratio, the removal rate of lignin gradually increased. When the solid–liquid ratio was 1:20, the removal rate of lignin was extremely low, which may be due to the fact that too little DES could not completely remove the lignin from the pulp [28]. When the solid–liquid ratio increases from 1:40 to 1:80, the lignin-removal rate increases slowly. Considering the cost of the DES, we chose the solid–liquid ratio of 1:40 as the best solid–liquid ratio for pulp treatment.

### 3.3. SEM Analysis

**Figure 5:**
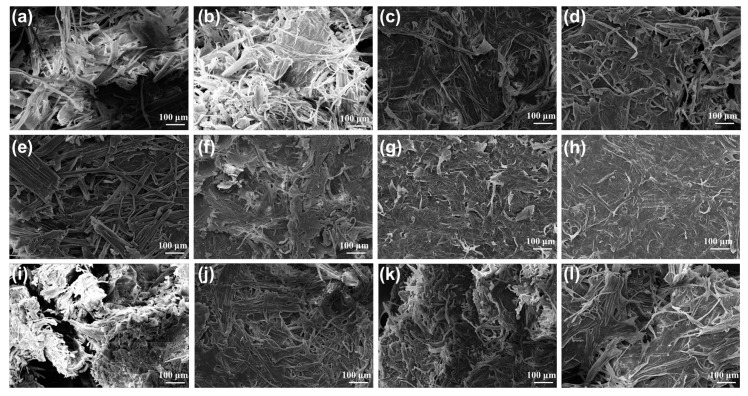
The SEM spectra of pulp after DES treatment under different processing conditions. Different times, including (**a**) 0.5 h, (**b**) 1 h, (**c**) 2 h, and (**d**) 6 h; different temperatures, including (**e**) 80 °C, (**f**) 110 °C, (**g**) 140 °C, and (**h**) 160 °C; and different solid–liquid ratios, including (**i**) 1:20, (**j**) 1:40, (**k**) 1:60, and (**l**) 1:80.

Figure 5 shows the SEM images of the slurry after DES treatment under different conditions. As the DES treatment time increased (Figure 5a–d), the outer surface of the slurry became rough, and the surface became uneven. The surface structure of cellulose becomes looser as the reaction time increases; the arrangement is disordered, and the pores become larger. Fiber length also shortens with time. This shows that as the reaction time increases, the effect of the DES on slurry treatment becomes more obvious. As the temperature increases, the fiber length becomes shorter, and there is no significant change in the fiber at 80 °C (Figure 5e). It can be seen from Figure 5g–h that when the temperature reaches 140 °C, almost no fiber exists, indicating that the high temperature will lead to serious degradation of cellulose, which is consistent with the results of component analysis. When the solid–liquid ratio increased from 1:20 to 1:40, it can be seen that the outline of the fiber was more obvious, which confirmed that most of the lignin was removed after DES treatment [38]. The morphology of cellulose did not change significantly with the increase in solid–liquid ratio (Figure 5j–l). This shows that further increasing the solid–liquid ratio has no obvious effect on the cellulose in the slurry.

### 3.4. FT-IR Analysis

**Figure 6:**
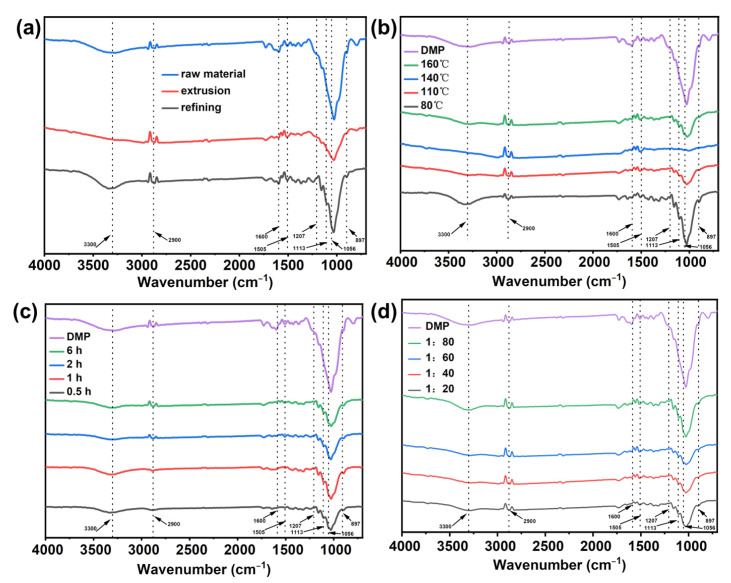
The FT-IR spectra of pulp: (**a**) raw material and after mechanical processing, (**b**) different temperatures, (**c**) different times, and (**d**) different solid–liquid ratios.

As shown in Figure 6, almost no peaks can be seen at 1207 cm^−1^ and 1056 cm^−1^ for the raw material after extrusion and after three-stage refining, but after extrusion and three-stage refining, the slurry has a peak at 1113 cm^−1^, and the peak at 897 cm^−1^ has increased, which may be due to the separation of fibers by mechanical treatment and the removal of some impurities increasing the peak intensity. However, after the deep eutectic solvent treatment, the characteristic peaks of cellulose did not disappear, indicating that the process of DES treatment of pulp would not change the functional groups of cellulose [40]. Among them, the broad absorption peak at 3200~3600 cm^−1^ is the stretching vibration peak of -OH; the stretching vibration peak of methine (C-H) is at 2900 cm^−1^. The characteristic peaks of lignin were significantly weakened, indicating that the process of DES treatment of pulp removed lignin in wheat straw pulp [21]. The peak located at 1239 cm^−1^ was attributed to the C–O–C stretching vibration peak of hemicellulose and lignin, 1505 cm^−1^ is the tensile vibration peak of the lignin aromatic skeleton, and 1600 cm^−1^ is the tensile vibration peak of the lignin aromatic skeleton and C=O. After treatment, the intensity of characteristic peaks is reduced, indicating that lignin is effectively removed [41,42]. The absorption peaks at 1113 cm^−1^, 1056 cm^−1^, and 897 cm^−1^ representing the characteristic peaks of cellulose are enhanced, which shows that after DES treatment, the proportion of cellulose in the pulp is increased compared with that of untreated pulp [43].

### 3.5. XRD Analysis

**Figure 7:**
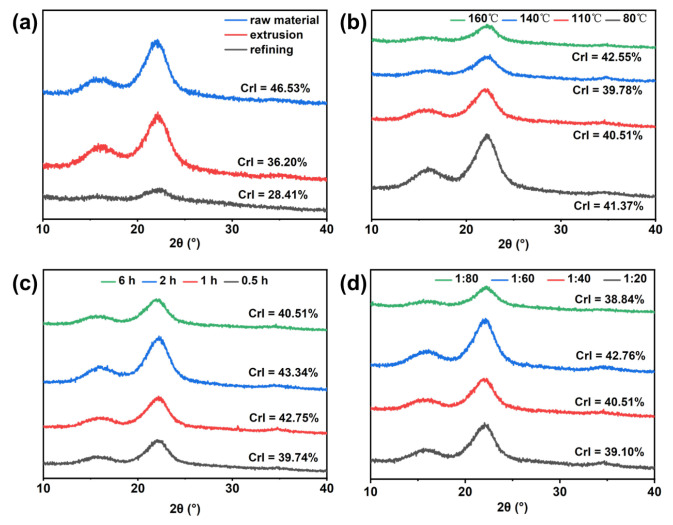
The XRD pattern of pulp: (**a**) raw material and after mechanical processing, (**b**) different temperatures, (**c**) different times, and (**d**) different solid–liquid ratios.

As shown in Figure 7, the XRD patterns of the raw material, mechanically treated pulp, and DES-treated pulp are similar, and they all have strong diffraction peaks around 2θ = 15.8° and 22.6°, which are consistent with the diffraction peaks of cellulose [44]. This shows that mechanical treatment and DES treatment did not change the crystal structure of cellulose. However, after mechanical treatment of wheat straw, the CrI value decreased significantly, which may be due to the relative weakening of the hydrogen bond force between wheat straw fibers due to the force generated by the mechanical treatment process, resulting in the destruction of a part of the crystallization area [45]. Compared with mechanical treatment, the CrI value of the pulp increased after DES treatment, and the CrI value was 40.51% under the optimal conditions of 110 °C, 1:40, and 6 h. The increase in pulp crystallinity may be attributed to the removal of lignin and the acid hydrolysis of the non-crystalline regions of cellulose promoted by acidic DES, which increased the proportion of crystalline regions [20].

### 3.6. DES Recovery Analysis

**Figure 8:**
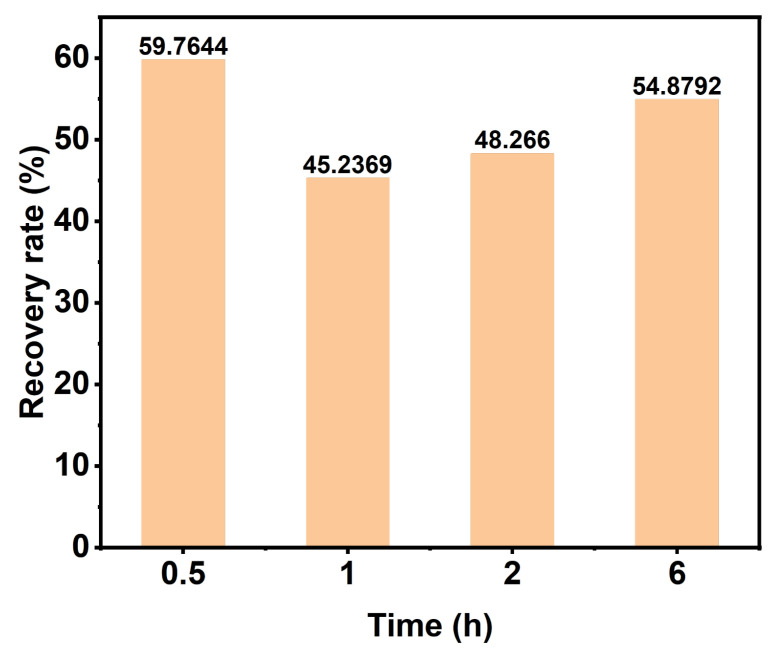
The DES recovery rate under different treatment times.

Compared to ionic liquids, a DES has the advantage of easier recyclability. This is because the recovery process only involves the formation or destruction of hydrogen bonds between the hydrogen bond acceptor (HBA) and the hydrogen bond donor (HBD), without any other chemical reactions [46]. We used a rotary evaporator to recover DESs with different reaction times and then detected the DES content by liquid chromatography and calculated the DES recovery rate, as shown in Figure 8. When the reaction time is 6 h, the DES recovery rate exceeds 50%, and a higher recovery rate can improve cost-effectiveness. Determining how to further improve the DES recovery rate in the future is a problem worthy of attention.

### 3.7. Pulp Bleaching

The pulp was bleached using an H&P two-stage bleaching process, and the whiteness performance of the pulp was characterized by measuring the whiteness of the paper. In addition, the viscosity, α-cellulose content, and Fock reactivity test results of the pulp before and after bleaching were measured. The results are shown in Table 1.

The bleaching of the pulp after DES treatment can effectively remove the residual lignin and significantly improve the whiteness of the pulp. An automatic pulp viscometer was used to test the measured pulp. The sample to be measured was tested twice in parallel, and the average value was taken as the viscosity of the pulp to be measured. The viscosity of the pulp after DES treatment was reduced from 623 mL/g to 472 mL/g. Moderate viscosity reduction is beneficial for the application of dissolved pulp in viscose rayon processing. As shown in Table 1, the content of alpha cellulose increased after bleaching (81.79% vs. 61.82%), demonstrating that bleaching can further remove lignin. The morphology of the pulp was characterized using a fiber quality analyzer. As can be seen from Table 2, the treated wheat straw pulp showed different degrees of fiber deformation, including kinking and curling. The fiber length of wheat straw pulp after bleaching was shorter than that before bleaching. It can also be seen from the table that the fines content of the pulp is high, which may be due to the fact that many fiber fragments are “detached” from the pulp fiber during the processing process, resulting in a high fines content of the pulp [28].

### 3.8. Mass Balance in the Preparation Process

**Figure 9:**
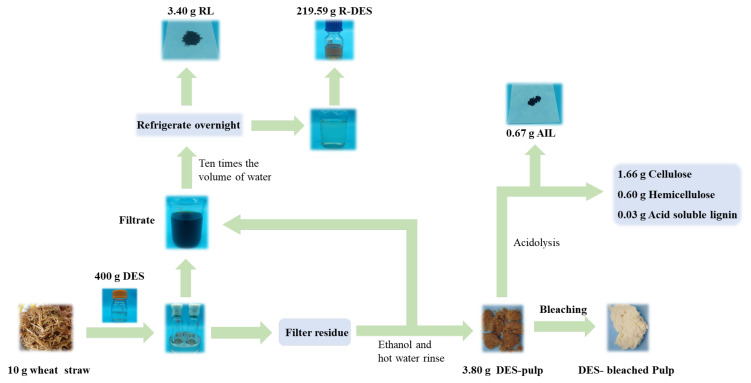
Schematic diagram of mass balance in the preparation process.

Figure 9 shows the schematic diagram of the process flow of preparing dissolved pulp by treating wheat straw raw material with a DES. The mass balance of each component of wheat straw was evaluated under the optimal conditions (110 °C, 1:40, 6 h). By treating 10 g of wheat straw raw material with the DES, 3.8 g of dissolved pulp was obtained and used for further treatment. In addition, 3.4 g of lignin was obtained by precipitation of waste liquid. In addition, close to 220 g of the DES was obtained by rotary evaporation treatment, which is conducive to the reuse of the DES. Based on the initial mass of wheat straw, the total recovery reached 72%, which is significantly higher than that in a previous study [47].

## 4. Conclusions

In this study, a process for the preparation of high-quality dissolving pulp was developed using wheat straw as a raw material. The results indicated that DES treatment increased the glucose and xylose content while reducing the lignin content in the slurry. The most effective removal of lignin was achieved at 110 °C, 6 h, and a solid–liquid ratio of 1:40. Compared to the raw material, the glucose content increased by 11.42%, while the acid-soluble lignin content and the acid-insoluble lignin content decreased by 2.18% and 21.6%, respectively. The DES recovery rate under this condition exceeded 50%. DES treatment did not cause chemical changes to the cellulose in the pulp. After further bleaching treatment, the α-cellulose content and Fock reactivity of the dissolved pulp were increased by 19.97% and 11.61%, respectively, and the viscosity was decreased by 151 mL/g. The lignin in wheat straw can be effectively removed by combined treatment with a mechanical deep eutectic solvent so that higher-quality wheat straw dissolving pulp can be produced. This research content has laid a certain research foundation for the resource utilization of the whole components of wheat straw.

## Figures and Tables

**Table 1:** Performance table of dissolved pulp before and after bleaching.

Pulp Classification	Brightness (%ISO)	Viscosity (mL/g)	α-Cellulose Content (%)	Fock Reactivity (%)
DES Pulp	28.64	623	61.82	44.24
DES Bleached Pulp	73.46	472	81.79	55.85

**Table 2:** Morphological properties of wheat straw pulp fiber.

Pulp Classification	Average Length (mm)			Fines Content (%)	Average Width (µm)	Curl (%)	Kink Angle (°)	Fibrillation (%)
	Lc(n)	Lc(l)	Lc(w)					
DES Pulp	0.52	0.82	1.32	86.76	29.81	5.21	37.84	1.74
DES Bleached Pulp	0.49	0.71	1.05	84.63	34.44	7.30	38.84	1.72

## Data Availability

The data presented in this study are available on request from the corresponding author.

## References

[1] Rabemanolontsoa H., Saka S. (2016). Various pretreatments of lignocellulosics. Bioresour. Technol..

[2] Dessbesell L., Paleologou M., Leitch M., Pulkki R., Xu C. (2020). Global lignin supply overview and kraft lignin potential as an alternative for petroleum-based polymers. Renew. Sustain. Energy Rev..

[3] Bourmaud A., Shah D.U., Beaugrand J., Dhakal H.N. (2020). Property changes in plant fibres during the processing of bio-based composites. Ind. Crop. Prod..

[4] Sadeghifar H., Venditti R., Jur J., Gorga R.E., Pawlak J.J. (2016). Cellulose-Lignin Biodegradable and Flexible UV Protection Film. ACS Sustain. Chem. Eng..

[5] Feng Y., Zhang D., Liang Y., Yin X., Lei B. (2021). A facile strategy for preparing lignocellulose-based bioplastic by grafting with quaternary ammonium salts. Ind. Crop. Prod..

[6] Chen J., Zhang B., Luo L., Zhang F., Yi Y., Shan Y., Liu B., Zhou Y., Wang X., Lü X. (2021). A review on recycling techniques for bioethanol production from lignocellulosic biomass. Renew. Sustain. Energy Rev..

[7] Singh G., Arya S.K. (2021). A review on management of rice straw by use of cleaner technologies: Abundant opportunities and expectations for Indian farming. J. Clean. Prod..

[8] Saini S., Kardam S.K., Kadam A.A., Kumar V., Gaikwad K.K. (2022). Green and energy-efficient extraction of cellulose nano-fibrils from rice straw and its coating to improve functional properties of rice straw paperboard made via refiner mechanical pulping. Prog. Org. Coat..

[9] Bajpai P. (2023). Concerns of the Conventional Pulping Methods. Environmentally Benign Pulping.

[10] Smith E.L., Abbott A.P., Ryder K.S. (2014). Deep eutectic solvents (DESs) and their applications. Chem. Rev..

[11] Shen X.-J., Wen J.-L., Mei Q.-Q., Chen X., Sun D., Yuan T.-Q., Sun R.-C. (2019). Facile fractionation of lignocelluloses by biomass-derived deep eutectic solvent (DES) pretreatment for cellulose enzymatic hydrolysis and lignin valorization. Green Chem..

[12] Wu X., Xiong J., Huang Z., Cao S., Zong M., Lou W. (2019). Improving biocatalysis of cefaclor with penicillin acylase immobilized on magnetic nanocrystalline cellulose in deep eutectic solvent based co-solvent. Bioresour. Technol..

[13] Liu M., Cao D., Bi W., Chen D.D.Y. (2021). Extraction of Natural Products by Direct Formation of Eutectic Systems. ACS Sustain. Chem. Eng..

[14] Sun Y., Cheng S., Mao Z., Lin Z., Ren X., Yu Z. (2020). High electrochemical activity of a Ti/SnO_2_-Sb electrode electrodeposited using deep eutectic solvent. Chemosphere.

[15] Kim J., Yoon Y., Kim S.K., Park S., Song W., Myung S., Jung H.K., Lee S.S., Yoon D.H., An K.S. (2021). Chemically Stabilized and Functionalized 2D—MXene with Deep Eutectic Solvents as Versatile Dispersion Medium. Adv. Funct. Mater..

[16] Wan Mahmood W.M.A., Lorwirachsutee A., Theodoropoulos C., Gonzalez-Miquel M. (2019). Polyol-Based Deep Eutectic Solvents for Extraction of Natural Polyphenolic Antioxidants from Chlorella vulgaris. ACS Sustain. Chem. Eng..

[17] Francisco M., van den Bruinhorst A., Kroon M.C. (2012). New natural and renewable low transition temperature mixtures (LTTMs): Screening as solvents for lignocellulosic biomass processing. Green Chem..

[18] Yu W., Wang C., Yi Y., Zhou W., Wang H., Yang Y., Tan Z. (2019). Choline chloride-based deep eutectic solvent systems as a pretreatment for nanofibrillation of ramie fibers. Cellulose.

[19] Colombo Dugoni G., Mezzetta A., Guazzelli L., Chiappe C., Ferro M., Mele A. (2020). Purification of Kraft cellulose under mild conditions using choline acetate based deep eutectic solvents. Green Chem..

[20] Duan C., Feng X., Tian C., Tian G., Nie S. (2023). Acidic deep eutectic solvent treatment for viscosity control and reactivity enhancement of bamboo dissolving pulp. Cellulose.

[21] Guo Z., Ling Z., Wang C., Zhang X., Xu F. (2018). Integration of facile deep eutectic solvents pretreatment for enhanced enzymatic hydrolysis and lignin valorization from industrial xylose residue. Bioresour. Technol..

[22] Duan C., Tian C., Feng X., Tian G., Liu X., Ni Y. (2023). Ultrafast process of microwave-assisted deep eutectic solvent to improve properties of bamboo dissolving pulp. Bioresour. Technol..

[23] Guo T., Yu Y., Wan Z., Zargar S., Wu J., Bi R., Sokhansanj S., Tu Q., Rojas O.J. (2022). Energy pellets from whole-wheat straw processed with a deep eutectic solvent: A comprehensive thermal, molecular and environmental evaluation. Renew. Energy.

[24] Yue X., Suopajarvi T., Sun S., Mankinen O., Mikkelson A., Huttunen H., Komulainen S., Romakkaniemi I., Ahola J., Telkki V.V. (2022). High-purity lignin fractions and nanospheres rich in phenolic hydroxyl and carboxyl groups isolated with alkaline deep eutectic solvent from wheat straw. Bioresour. Technol..

[25] Lou R., Ma R., Lin K.-t., Ahamed A., Zhang X. (2019). Facile Extraction of Wheat Straw by Deep Eutectic Solvent (DES) to Produce Lignin Nanoparticles. ACS Sustain. Chem. Eng..

[26] Suopajärvi T., Ricci P., Karvonen V., Ottolina G., Liimatainen H. (2020). Acidic and alkaline deep eutectic solvents in delignification and nanofibrillation of corn stalk, wheat straw, and rapeseed stem residues. Ind. Crop. Prod..

[27] Hou X.-D., Lin K.-P., Li A.-L., Yang L.-M., Fu M.-H. (2018). Effect of constituents molar ratios of deep eutectic solvents on rice straw fractionation efficiency and the micro-mechanism investigation. Ind. Crop. Prod..

[28] Chen Y., Shen K., He Z., Wu T., Huang C., Liang L., Fang G. (2021). Deep eutectic solvent recycling to prepare high purity dissolving pulp. Cellulose.

[29] Ji Q., Yu X., Yagoub A.E.-G.A., Chen L., Zhou C. (2020). Efficient removal of lignin from vegetable wastes by ultrasonic and microwave-assisted treatment with ternary deep eutectic solvent. Ind. Crop. Prod..

[30] Yu W., Yi Y., Wang H., Yang Y., Zeng L., Tan Z. (2021). Light-colored cellulose nanofibrils produced from raw sisal fibers without costly bleaching. Ind. Crop. Prod..

[31] Segal L., Creely J.J., Martin A.E., Conrad C.M. (1959). An Empirical Method for Estimating the Degree of Crystallinity of Native Cellulose Using the X-Ray Diffractometer. Text. Res. J..

[32] (1994). Fibrous Raw Material. Determination of Acid-Insoluble Lignin.

[33] Duan C., Li J., Ma X., Chen C., Liu Y., Stavik J., Ni Y. (2015). Comparison of acid sulfite (AS)- and prehydrolysis kraft (PHK)-based dissolving pulps. Cellulose.

[34] Tian C. (2013). Improvement in the Fock test for determining the reactivity of dissolving pulp. Tappi J..

[35] Jin R., Fan L., An X. (2011). Microwave assisted ionic liquid pretreatment of medicinal plants for fast solvent extraction of active ingredients. Sep. Purif. Technol..

[36] Song W., Deng Y., Zhu H., Bolf N. (2016). Research on Wheat Straw Pulping with Ionic Liquid 1-Ethyl-3-Methylimidazole Bromide. Kem. U Ind..

[37] Sannigrahi P., Kim D.H., Jung S., Ragauskas A. (2011). Pseudo-lignin and pretreatment chemistry. Energy Environ. Sci..

[38] Lim W.-L., Gunny A.A.N., Kasim F.H., AlNashef I.M., Arbain D. (2019). Alkaline deep eutectic solvent: A novel green solvent for lignocellulose pulping. Cellulose.

[39] Liu Q., Yuan T., Fu Q.-J., Bai Y.-Y., Peng F., Yao C.-L. (2019). Choline chloride-lactic acid deep eutectic solvent for delignification and nanocellulose production of moso bamboo. Cellulose.

[40] Montoya-Escobar N., Ospina-Acero D., Velasquez-Cock J.A., Gomez-Hoyos C., Serpa Guerra A., Ganan Rojo P.F., Velez Acosta L.M., Escobar J.P., Correa-Hincapie N., Triana-Chavez O. (2022). Use of Fourier Series in X-ray Diffraction (XRD) Analysis and Fourier-Transform Infrared Spectroscopy (FTIR) for Estimation of Crystallinity in Cellulose from Different Sources. Polymers.

[41] El Achaby M., Ruesgas-Ramón M., Fayoud N.-E.H., Figueroa-Espinoza M.C., Trabadelo V., Draoui K., Ben Youcef H. (2019). Bio-sourced porous cellulose microfibrils from coffee pulp for wastewater treatment. Cellulose.

[42] Achinivu E.C. (2018). Protic Ionic Liquids for Lignin Extraction-A Lignin Characterization Study. Int. J. Mol. Sci..

[43] Kesari K.K., Leppänen M., Ceccherini S., Seitsonen J., Väisänen S., Altgen M., Johansson L.S., Maloney T., Ruokolainen J., Vuorinen T. (2020). Chemical characterization and ultrastructure study of pulp fibers. Mater. Today Chem..

[44] Fatah I., Khalil H., Hossain M., Aziz A., Davoudpour Y., Dungani R., Bhat A. (2014). Exploration of a Chemo-Mechanical Technique for the Isolation of Nanofibrillated Cellulosic Fiber from Oil Palm Empty Fruit Bunch as a Reinforcing Agent in Composites Materials. Polymers.

[45] He M., Yang G., Chen J., Ji X., Wang Q. (2018). Production and Characterization of Cellulose Nanofibrils from Different Chemical and Mechanical Pulps. J. Wood Chem. Technol..

[46] Xu P., Zheng G.W., Zong M.H., Li N., Lou W.Y. (2017). Recent progress on deep eutectic solvents in biocatalysis. Bioresour. Bioprocess.

[47] Chen Y., Yan Z., Liang L., Ran M., Wu T., Wang B., Zou X., Zhao M., Fang G., Shen K. (2020). Comparative Evaluation of Organic Acid Pretreatment of Eucalyptus for Kraft Dissolving Pulp Production. Materials.

